# Rapid Microfluidic Immunoassays of Cancer Biomarker Proteins Using Disposable Inkjet-Printed Gold Nanoparticle Arrays

**DOI:** 10.1002/open.201300018

**Published:** 2013-06-12

**Authors:** Colleen E Krause, Brunah A Otieno, Alina Latus, Ronaldo C Faria, Vyomesh Patel, J Silvio Gutkind, James F Rusling

**Affiliations:** [a] Department of Chemistry, University of ConnecticutStorrs, CT 0626 (USA) E-mail: james.rusling@uconn.edu; [b] Institute of Physical Chemistry, “I. Murgulescu” Romanian Academy Splaiul, Independentei 202Bucarest 060021 (Romania); [c] Departamento de Quimic, Universidade Federal de Sao CarlosSao Paulo (Brazil); [d] Pharyngeal Cancer Branch, National Institute of Dental and Craniofacial Research, National Institutes of HealthBethesda, MD (USA); [e] Department of Cell Biology, University of Connecticut, Health CenterFarmington, CT 0623 (USA); [f] Department of Chemistry, National University of Ireland at GalwayGalway (Ireland)

**Keywords:** cancer biomarkers, electrochemistry, inkjet-printed arrays, microfluidics, sensors

Conventional protein detection methods such as enzyme-linked immunosorbent assays (ELISA) often take many hours to complete and usually only apply to one protein at a time. More rapid, multiplexed methods are needed for point-of-care (POC) and surgical applications in future personalized cancer diagnostics and therapy. This paper describes a low-cost inkjet-printed gold nanoparticle (AuNP) sensor chip integrated into a simple microfluidic immunoarray to achieve detection of two cancer biomarker proteins in 5 μL samples in 8 min. Magnetic beads of 1 μm diameter derivatized with ∼300 000 enzyme labels and thousands of antibodies were used to capture the biomarker proteins from samples. The beads with captured proteins are then injected into the microfluidic system and captured by antibodies on nanostructured sensor elements to provide high sensitivity and ultralow detection limits (DL). For assay times of 45 mins, DLs were 78 fg mL^−1^ for interleukin-6 (IL-6) and 19 fg mL^−1^ for interleukin-8 (IL-8). Decreasing assay time to 8 min provided clinically relevant DLs of 5 pg mL^−1^. Accuracy was demonstrated by determining IL-6 and IL-8 in conditioned media from head and neck squamous cell carcinoma (HNSCC) cells and comparing results to those from standard single-protein ELISAs. Results suggest that this device can be employed for rapid detection of a wide range of disease-related proteins in clinical applications.

Despite recent advances in treatment, cancer remains a leading worldwide cause of human mortality. Current methods of cancer detection are often based on imaging technologies, such as magnetic resonance imaging (MRI), positron emission tomography (PET), and computed tomography (CT), which themselves have improved in performance using new contrast materials and can distinguish between different anatomical features.[1–5] However, these approaches rely on finding and imaging a tumor, giving limited information on the onset of cancer or quantification of cancerous cells.[1–5] Other techniques are based on cell morphology and microscopy, which involve invasive biopsies to observe cancer cells in tissue.[6, 7] These tests are not individually conclusive, as biopsies can miss concentrations of cancer cells, especially at early stages of the disease.[6, 7] Alternatively, specific biosensor arrays that rapidly measure multiple biomarker proteins in serum provide hope for future early cancer detection and monitoring.[8–14] Such sensitive detection schemes for a selective protein panel whose members are elevated at the onset of cancer are expected to greatly improve patient prognoses and treatment outcomes and may even lead to cancer prevention.[15] Immunosensor microarrays show great potential in targeting specific biomarkers especially when integrated with microfluidics.[15]

IL-6 and IL-8 were chosen as test biomarkers in this study. These pro-inflammatory cytokines influence all stages of tumor development including initiation, progression and metastasis.[16, 17] IL-6 and IL-8 have been used to detect and monitor HNSCC commonly referred to as oral cancer.[18, 19] HNSCC has high mortality rates due to late diagnosis based on current methods, mainly relying on visual identification of cancerous lesions.[20, 21] Serum levels of IL-6 in patients with oral cancer are commonly ≥20 pg mL^−1^, whereas healthy individuals are below 6 pg mL^−1^.[22] Similarly, IL-8 serum levels in oral cancer patients are usually above 20 pg mL^−1^, with concentrations below 13 pg mL^−1^ observed in healthy individuals.[23] A biosensor for IL-8 in serum was developed by Munge et al.[24] with a DL of 1 fg mL^−1^ in ∼1 hour assay time. However, there is a need to rapidly measure multiple biomarker proteins in surgical applications to inform decisions such as defining surgical borders and metastasis and to detect and monitor recurrence.[25]

Effective POC sensors must be inexpensive, rapid, adequately sensitive, and should require limited technical expertise and minimal sample volume.[8, 9] A number of methods including fluorescence immunoassays,[27] PCR-based bar code labels,[26] radioimmunoassay,[28] 2D electrophoresis,[29] and multidimensional liquid chromatography-mass spectrometry[30] have been used, but most are limited for POC protein measurements due to cost, assay time, or technical complexity. Recent approaches to decrease assay times include an immuno-pillar chip[31] which gives immunoassay in 4 min for C-reactive protein using fluorescence detection.[31] Here, a 3D hydrogel format impeded removal of nonspecifically bound antibodies in wash steps, the assay yielded relatively high DLs of 100 pg mL^−1^ for C-reactive protein and ng mL^−1^ levels for α-fetoprotein and prostatespecific antigen (PSA) in serum.[31, 32]

We recently fabricated disposable inkjet-printed arrays from 4 nm AuNPs that cost less than $ 0.20 per array in materials.[33] Preliminary tests of these gold arrays without microfluidics achieved a DL of 20 pg mL^−1^ for IL-6. To facilitate rapid, semi-automated measurements of proteins in the present work, these AuNP arrays were integrated into a simple microfluidic device, and protein analytes were captured for analysis with magnetic beads (MB) bioconjugated with hundreds of thousands of enzyme labels and secondary antibodies (Ab_2_, see Scheme 1).[15] We have also used sensor arrays made from commercial screen-printed carbon arrays decorated with 5 nm AuNPs in a microfluidic system to achieve 1.25 hour assays with DLs of 200 fg mL^−1^ for IL-6 and PSA in serum.[15] We later improved DLs to 5–50 fg mL^−1^ for determining four oral cancer biomarker proteins in serum simultaneously in 50 min assays by using 400 000 horseradish peroxidase (HRP) labels per bead.[18] The disadvantage here is that the disposable sensor arrays cost about $ 10 each and require manual deposition of AuNPs onto a polyion film on each sensor in the array. The advantage of the printed AuNP immunoarrays in this paper is that they are printed directly on thermostable plastic at low cost with automatic production of hundreds of arrays at a time using an inkjet materials printer.[33] Here we describe integration of this low-cost AuNP immunoarray into a microfluidic device to achieve clinically relevant DLs of ∼5 pg mL^−1^ for IL-6 and IL-8 in 8 min.

**Scheme 1:**
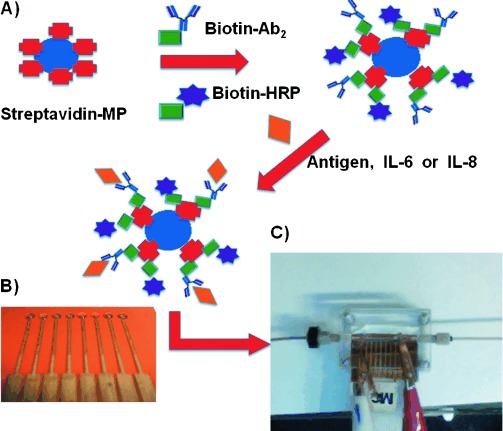
A) Off-line analyte protein capture on Ab_2_-MB-HRP magnetic beads (MB) in a small vial. B) Inkjet-printed AuNP immunoarray, sensors are spots at the top; contacts are rectangular pads at bottom. C) Immunoarray in the microfluidic detection chamber where antibody-decorated sensors capture Ab_2_-MB-HRP-protein conjugates. The array is connected to a syringe pump and sample valve for operation.

Arrays were printed with ink made from toluene and 4 nm alkylthiol-protected AuNPs on Kapton polymer sheets, annealed for 3 min at 200 °C to sinter particles and remove thiols, then insulated with a printed Kapton layer (see Supporting Information).[19, 33, 34] Arrays were cleaned by cycling potential between 1.5 and −0.2 V vs. saturated calomel electrode (SCE) in 0.18 m sulfuric acid to remove gold oxide (Supporting Information, Figure S6 A). Then, a self-assembled monolayer (SAM) of 3-mercaptopropionic acid (MPA) was chemisorbed onto sensor surfaces to provide surface carboxyl groups. These carboxyl groups were then activated by *N*-(3-dimethylaminopropyl)-*N′*-ethylcarbodiimide hydrochloride (EDC) and *N*-hydroxysulfosuccinimide (NHSS), and primary antibodies (Ab_1_) were attached to the array by amidization.

Prior to incorporating the array into the microfluidic device, arrays with Ab_1_ attached were washed with phosphate buffered saline (PBS; pH 7, 0.05 % Tween-20) to remove unreacted Ab_1_. Arrays were then incubated outside the microfluidic system with 2 % bovine serum albumin (BSA) to decrease subsequent nonspecific binding. All assay parameters, including concentrations and incubation times, were optimized for high sensitivity and high signal-to-noise ratio.

Ab_2_-MB-HRP beads were first synthesized starting with 1 μm tosylated MBs for off-line capture as reported previously.[15] These Ab_2_-MB-HRP bioconjugates had 110 000 (±20 000) copies of Ab_2_ and 130 000 (±35 000) HRPs (Supporting Information, Table S1). Different beads were used to capture IL-6 and IL-8 from the samples. IL-6 and IL-8 in undiluted calf serum were captured off-line in a vial by these heavily labeled antibody-equipped magnetic bead conjugates. Once the beads captured their specific proteins, the bioconjugates were separated magnetically from the test solutions, washed, and redispersed in buffer. This dispersion was used to fill the sample loop in the injection valve, and then injected into the microfluidic detection chamber housing the 8-sensor AuNP array. The flow was stopped when the beads filled the chamber, as indicated by their red-brown color, and an incubation period was allowed for primary antibodies on sensor surfaces to capture the bioconjugate beads. This was followed by again washing with PBS (pH 7, 0.05 % Tween-20).

To generate amperometric responses, a mixture of 1 mm hydroquinone (HQ) mediator and 0.1 mm H_2_O_2_ was injected into the microfluidic device via the 100 μL sample loop with potential at −0.2 V vs. Ag/AgCl.[15] H_2_O_2_ activates the iron heme protein HRP to the ferryloxy form, which is reduced by electron mediator HQ that accepts electrons from the sensor. Control experiments featured the full immunoassay procedure without IL-6 and IL-8. For optimum sensitivity, we first used a total assay time of 45 min, with 25 min incubation for off-line capture of antigens, a second incubation of beads in the detection chamber for 15 min, and wash steps and detection <4 min. Individual sensor areas were determined and signals are reported as current densities.

In the 45 min assays (Figure 1), peak current densities increased logarithmically from 160 fg mL^−1^ to 3750 fg mL^−1^ for IL-6 with a DL of 160 fg mL^−1^. DLs were determined as the signal three standard deviations above that of the control. Representative amperometric responses are shown in Figure 1 A, and a calibration curve is shown in Figure 1 B, documenting high sensitivity in the fg mL^−1^ range and below.

**Figure 1:**
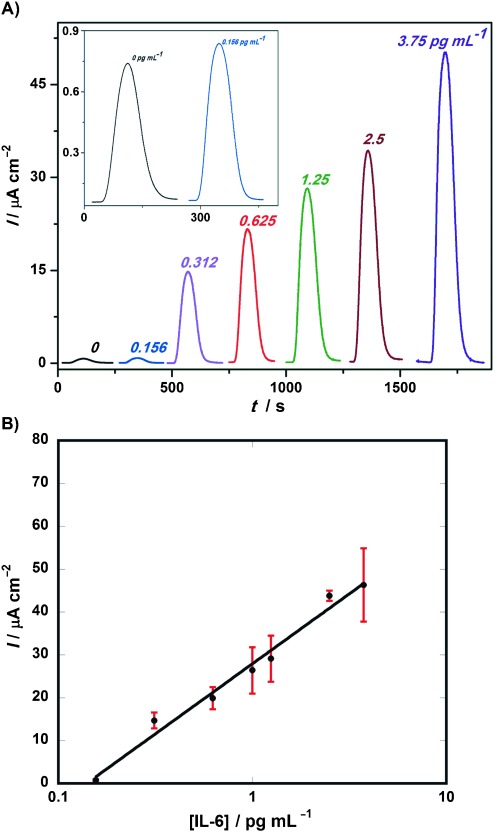
A) Amperometric responses of IL-6 in undiluted calf serum at −0.2 V vs. Ag/AgCl developed by injecting 1 mm HQ and 0.1 mm H_2_O_2_ after capturing analyte-Ab_2_-MB_tosyl_-HRP bioconjugates on the sensors in the microfluidic device. Inset shows lowest level and control on expanded current scale. B) Corresponding calibration curve *y*=27.9+32.7 log(*x*); *R*=0.99.

Attachment of Ab_2_ and HRP to tosylated MBs involves simple mixing of the reactants, and proteins become attached by secondary amine linkages. However, preparation takes 42 h, so we also evaluated streptavidin-coated magnetic beads with a conjugate preparation time of 25 min. These Ab_2_-MB-HRP bioconjugates had an estimated ∼38 000 (±7000) copies of Ab_2_ and ∼320 000 (±23,000) HRPs (Supporting Information, Table S1), and different beads were prepared to capture IL-6 and IL-8. Calibration data (Figure 2) showed that these streptavidin Ab_2_-MB-HRP bioconjugates gave peak current densities increasing logarithmically from 39 to 2500 fg mL^−1^ for IL-6 and a DL of 78 fg mL^−1^. A decrease in sensitivity was found from the slopes of the calibration plots for IL-6 when comparing slopes in Figure 1 and 2, although sensitivity is still quite adequate for clinical measurements. The much smaller conjugate preparation time outweighed the sensitivity decrease, and so we proceeded with further development of the streptavidin bead approach.

**Figure 2:**
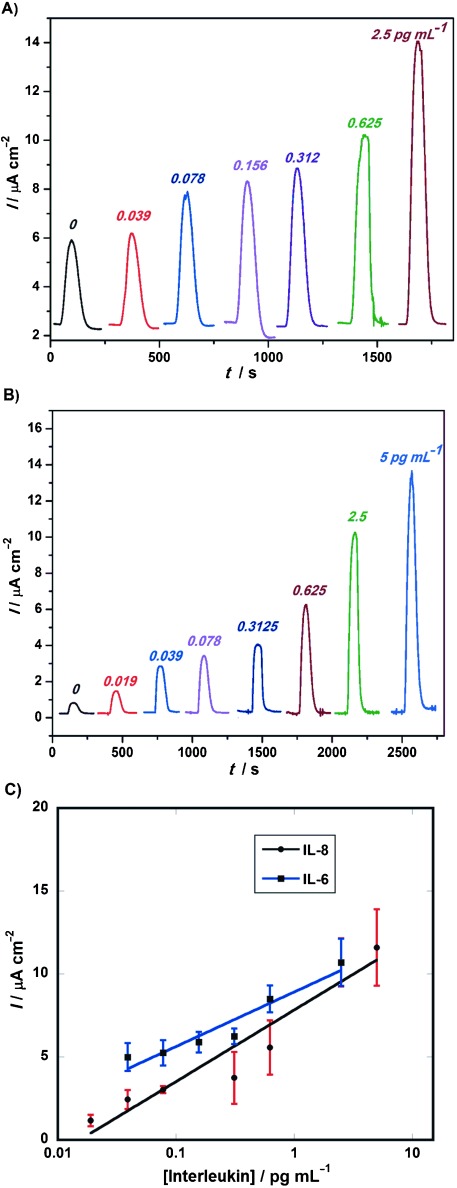
Amperometric peaks of A) IL-6 and B) IL-8 in calf serum mixtures in high sensitivity 45 min assays at −0.2 V vs. Ag/AgCl developed by injecting 1 mm HQ and 0.1 mm H_2_O_2_ after capturing analyte protein-Ab_2_-MB-HRP conjugates on the sensors in the microfluidic device. C) Corresponding calibration curves for IL-6 (blue), *y*=8.91+3.29 log(*x*); *R*=0.96, and for IL-8 (black), *y*=7.84+4.31 log(*x*); *R*=0.96.

The calibration curve for IL-8 in undiluted serum was similar to that of IL-6 with the streptavidin MBs. As before, we kept the concentration of Ab_1_ at 100 μg mL^−1^ and concentration of Ab_2_ at 20 μg mL^−1^. Peak current densities increased logarithmically from 19 to 5000 fg mL^−1^ for IL-8 with a DL of 19 fg mL^−1^. Representative amperometric responses are shown in Figure 2 B,C. The slopes of the calibration plots for both IL-6 and IL-8 indicated similar sensitivities for both biomarkers.

We then decreased assay time by sacrificing the ultralow detection limits for speed, with the aim of achieving dynamic ranges that match clinically significant concentrations in serum. Total assay time was measured from the time when IL-6 and IL-8 samples or standards were added to the MB capture reagent. Off-line capture was shortened to 3 min, the incubation of MBs with captured proteins in the detection chamber was 3 min, and washing and detection was <2 min (Supporting Information, Figure S8 A (IL-6) and Figure S8 B (IL-8)). Thus, total assay time was 8 minutes.

Using this time protocol, we obtained calibration curves of IL-6 and IL-8 in undiluted calf serum with concentrations of Ab_1_ and Ab_2_ as previously described. Representative amperometric peaks of biomarker proteins in 8 min assays are shown in Figure 3 A (IL-6) and Figure 3 B (IL-8), along with corresponding calibration curves (Figure 3 C). We found DLs of ∼5 pg mL^−1^ for IL-6 and IL-8, which is within the clinical normal range in serum for both proteins.[22, 23] Peaks increased with concentration from 5 pg mL^−1^ to 200 pg mL^−1^ for both proteins. Slopes of calibration curves (Figure 3 C) confirm that high sensitivity is achieved despite the short assay times. Comparing slopes for the 8 min assay data in Figure 3 C to that of the 45 min assay (Figure 2 C), there is only slightly lower sensitivity. For example, 8 min assays gave a sensitivity for IL-6 of 2.18 μA cm^−2^(log *c*)^−1^ compared to 3.29 μA cm^−2^(log *c*)^−1^ in a 45 min assay.

**Figure 3:**
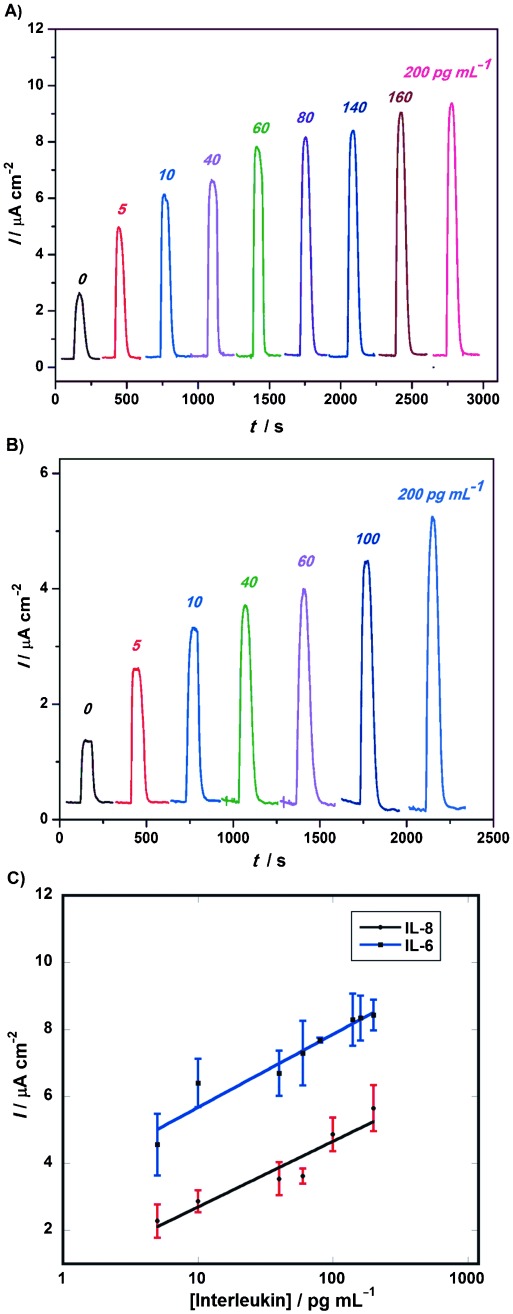
Amperometric peaks for A) IL-6 and B) IL-8 in calf serum mixtures in 8 min assays, at −0.2 V vs. Ag/AgCl developed by injecting 1 mm HQ and 0.1 mm H_2_O_2_ after capturing analyte protein Ab_2_-MB-HRPconjugates on the sensors in the microfluidic device. C) Corresponding calibration curves for IL-6 (blue), *y*=3.49+2.18 log(*x*); *R*=0.96, and for IL-8 (black), *y*=0.74+1.96 log(*x*); *R*=0.95.

Levels of human IL-6 and IL-8 in conditioned media secreted from oral cancer cell cultures were measured to validate accuracy. Excellent correlations were obtained between 8 min assays by the immunoarray and conventional ELISA (Figure 4). All of the representative HNSCC cell lines used (HN12, HN13, and Cal 27) secreted relatively large amounts of the biomarkers when compared to non-cancer counterpart cells (HaCaT). Linear correlation plots for the immunoarray versus ELISA gave slopes close to 1.0 and intercepts within standard deviation of zero, confirming strong correlation between the immunoarray and ELISA (Supporting Information, Figure S9).

**Figure 4:**
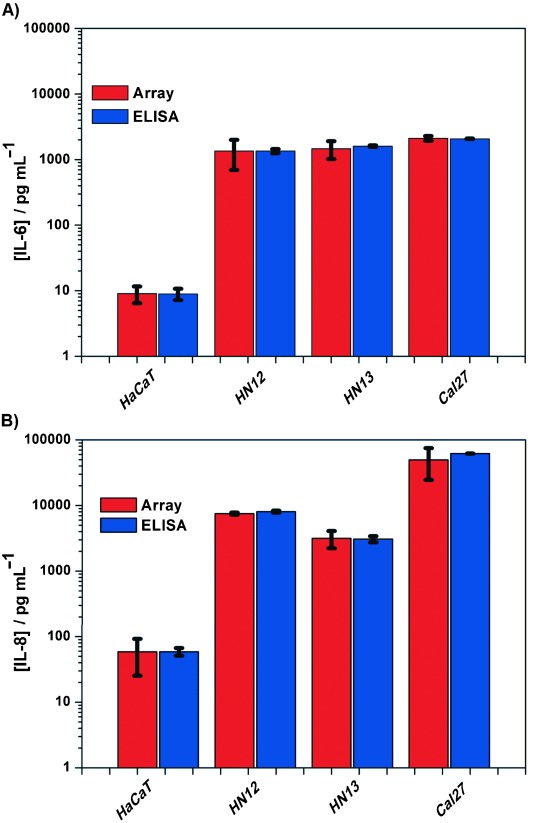
Comparison of immunoarray results with standard ELISA for conditioned media for cells (HaCaT, HN12, HN13, and Cal 27) for both A) IL-6 and B) IL-8.

Results described above demonstrate successful integration of a disposable, inkjet-printed AuNP immunoarray into a simple microfluidic device for multiple protein determinations. The novelty of the approach using off-line capture of proteins on heavily labeled magnetic beads for signal amplification allows optimization for either ultrasensitive detection or rapid clinical assays in 5 μL of serum. The AuNP immunoarray offers many promising features for rapid point-of-care applications, including low cost, high sensitivity, and multiplexing, but the current approach still requires moderate technical expertise. Operational features involving off-line capture, washing and reagent addition need to be simplified further, and we are currently addressing these issues in our laboratory.

## Experimental Section

Experimental procedures and materials are presented in detail in the Supporting Information.

Electrochemical measurements were made using a CHI 1040A eight-channel potentiostat (CH Instruments, Austin, TX, USA) at RT. Amperometry was done at optimal conditions for high sensitivity and low noise, −0.2 V vs. Ag/AgCl (0.14 m NaCl) at a flow rate of 100 μL min^−1^. The microfluidic device was constructed as previously reported[15] and featured a syringe pump, injector valve, and 60 μL polymdimethylsiloxane (PDMS) channel that housed sensors, reference, and counter electrodes.

## Supplementary material

As a service to our authors and readers, this journal provides supporting information supplied by the authors. Such materials are peer reviewed and may be re-organized for online delivery, but are not copy-edited or typeset. Technical support issues arising from supporting information (other than missing files) should be addressed to the authors.
