# Supplementary material for: Rapid Microfluidic Immunoassays of Cancer Biomarker Proteins Using Disposable Inkjet-Printed Gold Nanoparticle Arrays
Source: ChemistryOpen. 2013 Jun 12;2(4):141–5. doi: 10.1002/open.201300018 (PMC3775520; doi:10.1002/open.201300018)
Supplement: Supplementary file 1 [file open0002-0141-SD1.pdf]

ChemistryOPEN

Supporting Information

© 2013 The Authors. Published by Wiley-VCH Verlag GmbH & Co. KGaA, Weinheim

Rapid Microfluidic Immunoassays of Cancer Biomarker Proteins Using Disposable Inkjet-Printed Gold Nanoparticle Arrays

Colleen E. Krause,[a] Brunah A. Otieno,[a] Alina Latus,[a, b] Ronaldo C. Faria,[c] Vyomesh Patel,[d] J. Silvio Gutkind,[d] and James F. Rusling*[a, e, f]

open_201300018_sm_miscellaneous_information.pdf

Materials. Kapton FPC film (127 mM thick) was purchased from American Durafilm. Lyophilized 99% bovine serum albumin (BSA), sterile-filtered bovine calf serum, *gold (III) chloride trihydrate*, *1-dodecane thiol*, *tetrocylammonium bromide*, *sodium borohydride*, *3-mercaptopropionic acid* (MPA), *N-hydroxysulfosuccinimide* (NHSS), 1-*Ethyl-3-[-3-dimethylaminopropyl] carbodimmide hydrochloride* (EDC), *poly(amic acid)*, *Tween-20*, and *Hydroquinone* (HQ) were purchased from Sigma-Aldrich. *Hydrogen peroxide* (H_2O_2, 30%) came from Fisher Scientific. The *poly(dimethoxy)silane* (PDMS) kit was purchased from Dow Corning. MyOne *tosylactivated* beads (1 μm diameter, Dynabeads) and *streptavidin*-coupled magnetic beads (1 μm diameter, Dynabeads) were from Invitrogen. Immunoreagents (monoclonal primary antibodies, BSA, *biotinylated* secondary antibodies and *biotinylated* horseradish peroxidase (HRP)) were dissolved in pH 7.2 *phosphate saline* (PBS) buffer (5.9 mM Na_2HPO_4, 3.9 mM NaH_2PO_4, 2.7 mM KCl, 120 mM NaCl). 400 mM EDC and 100 mM NHSS were dissolved in ultrapure water immediately before use. Monoclonal antihuman Interleukin-6 (IL-6) antibody (Ab_1) (clone no. 6708), biotinylated antihuman IL-6 antibody (biotin-Ab_2), recombinant human IL-6 (carrier-free), human IL-6 polyclonal (goat IgG) antibody (Ab_2), monoclonal antihuman interleukin-8 (IL-8) antibody (AB_1) (clone no. 6217), biotinylated antihuman IL-8 antibody(biotin-Ab_2), recombinant human IL-8 (carrier-free) were from R&D Systems, Inc. (Minneapolis, MN, USA).

Microfluidic Device. The microfluidic system was constructed as previously reported by Chikkaveeraiah *et. al*. [1] It featured a molded, soft PDMS slab with a 1.5 mm wide rectangular channel that was placed on top of the electrode array. As before, the microfluidic channel was supported by two hard flat *poly(methylmethacrylate*) (PMMA) plates manufactured to fit on either side of the PDMS slab and bolted together to provide a sealed microfluidic channel 1.5 mm wide, 2.8 cm long and 63 μL in volume. [1] The top PMMA plate contained female ports (4 mm diameter) for screwing in standard male plastic fittings (1.5 mm, up church) to hold 0.2 mm i.d. PEEK connecting tubing for an inlet and outlet. The top PMMA plate also contained holes that were 0.6 mm for Ag/AgCl and 0.2 mm diameter for Pt wire electrode. For the microfluidic system, a Harvard 70-4504 pump 11 elite model syringe pump was used. It was connected to the inlet through a Rheodyne model 9725i injector valve with a sample loop of 100 μL through 0.2 mm i.d. tubing.

Characterization of Dodecanethiol Au-nanoparticles by Transmission Electron Microscopy (TEM). Dodecanethiol-protected gold nanoparticles were synthesized according to Jensen *et al*.[2] 200 mg of *dodecanethiol* Au-nanoparticles was dispersed in toluene at a concentration of 100 mg mL^{-1}. This solution was then diluted to a final concentration of 2 mg mL^{-1} for TEM imaging. TEM sample was prepared by placing one drop of the final dispersion on a silica wafer and drying under vacuum. The average diameter was 4 (± 2) nm shown in Figure S1.

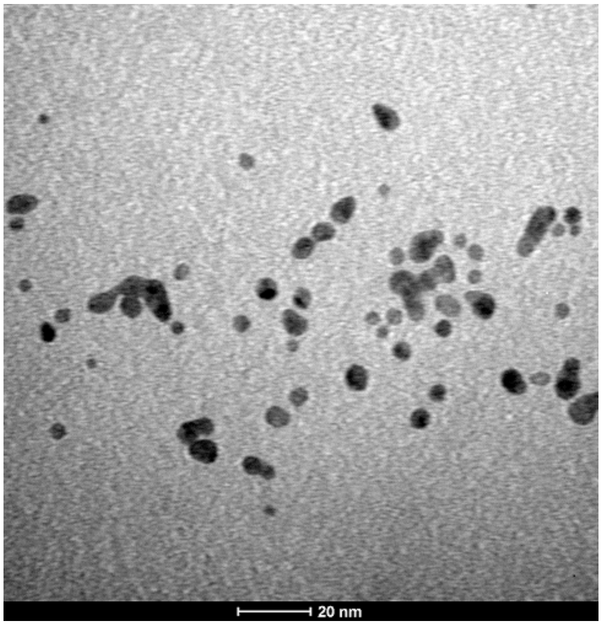

Figure S1: TEM image of dodecanethiol gold-nanoparticles with average diameters of 4 ± 2 nm on 20 nm scale bars.

Gold Array Fabrication and Characterization. Gold arrays were fabricated as described previously by Jensen *et al*. using a Dimatrix Inkjet materials printer.[2] The gold nanoparticle ink was prepared at a concentration of 100 mg mL^{-1} in *toluene* and filtered with a 0.2 mm cutoff PTFE filter. The ink was injected into a liquid crystal Dimatix printer cartridge immediately prior to use.[2] Once the gold was printed, the arrays were then sintered for 15 mins at 200°C.[2] The arrays lightened in color indicating loss of the *dodecane thiol* layer and Au cores coalescing. [3] The reduction of thiols by 43% was confirmed by EDX (Figure S2, S3), as well as by FESEM micrographs (Figure S4). The arrays were then immediately returned to the Inkjet materials to print the insulation layer of *poly(amic acid)*. The *poly(amic acid)* ink was also prepared, as previously reported,[2] by diluting the 10% (m/m) poly(amic acid) solution in highly pure *N-methyl-2-pyrrolidone* (NMP) to 1% (m/m) and added to a liquid crystal Dimatix printer cartridge immediately prior to use.

Energy-dispersive X-Ray Spectroscopy, EDX elemental scan confirmed the loss of *dodecanethiol* layer.[3] The elemental scan before sintering is shown in Figure S2 with percentage of *sulfur* at 3.3%. The elemental scan after sintering is shown in Figure S3 with percentage of sulfur at 1.4% indicating a loss of ~43% once the arrays are baked.

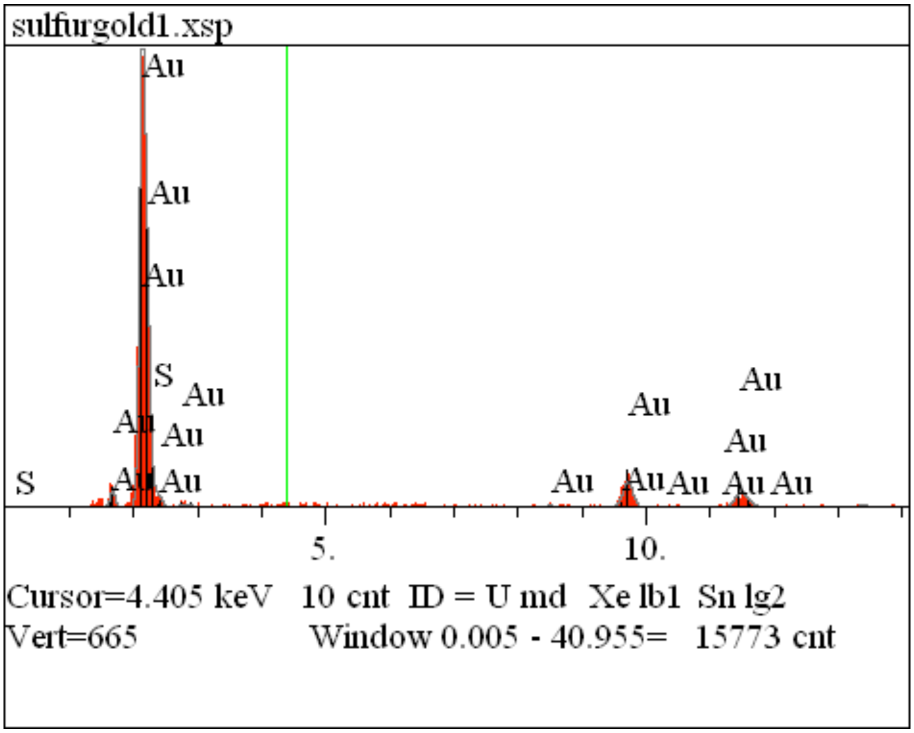

Figure S2: EDX pre-sintering elemental scan.

Elt.	Line	Atomic %	Conc	Units	
S	Ka	17.302	3.294	wt.%	
Au	La	82.698	96.706	wt.%	
		100.000	100.000	wt.%	Total

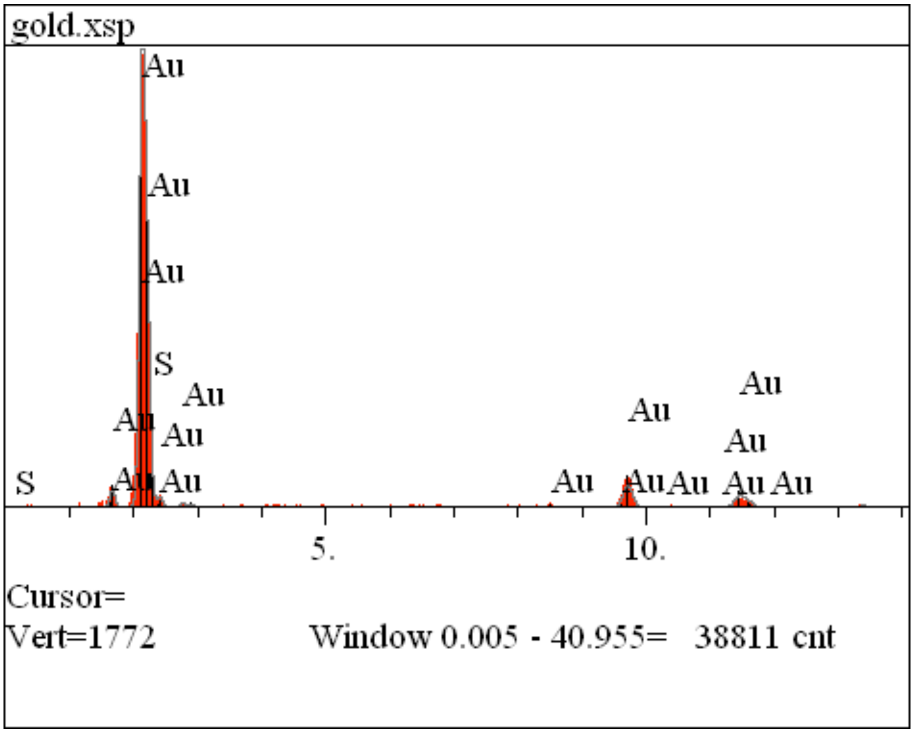

Figure S3: EDX post-sintering elemental scan.

Elt.	Line	Atomic %	Conc	Units	
S	Ka	8.145	1.423	wt.%	
Au	La	91.855	98.577	wt.%	
		100.000	100.000	wt.%	Total

Field Emission Scanning Electron Microscopy, FESEM confirmed formation of percolated paths due to the loss of dodecanethiol layer. Scanning Electron Micrographs were taken on a Zeiss DSM 982 Gemini FE-SEM with a Schottky Emitter at an accelerating voltage range from 2 to 4 kV and a beam current of about 1 mA. Printed *dodecanethiol* before sintering is shown in figure S2A indicating large gaps between the inkjet-printed scans that when heat treated at 200°C for 15 mins were removed due to the loss of *dodecanethiol* groups forming the percolated paths as the Au cores coalesced as shown in Figure S4B and at the same magnification as figure S4A. It seems as though there are cracks in Figure S4B but on further magnification we can see that they are just differences in the thickness of layers of the sintered gold (Figure S4C).

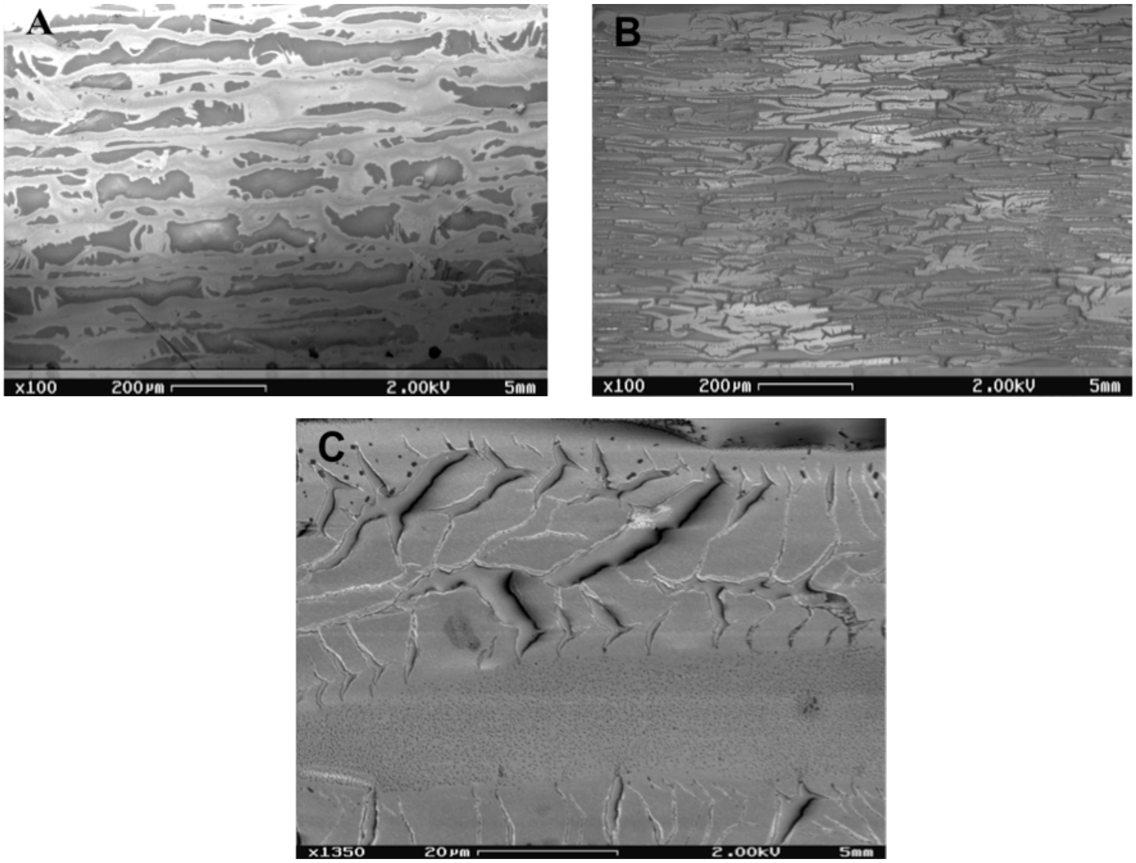

Figure S4: FESEM micrograph of an electrode on an array before sintering (A) and after (B) indicating that the Au cores coalesced, then a larger magnification (C) after sintering to demonstrate that it's not cracking but different thickness of films.

Electrochemical Properties of Inkjet-printed Gold Arrays. To assess the reproducibility of the arrays, cyclic voltammetry was done on all eight electrodes simultaneously in an unstirred solution of 1 mM potassium ferricyanide solution in 100 mM potassium chloride in the microfluidic device between -0.2 V to 0.6 V at 100 mV s^{-1}.

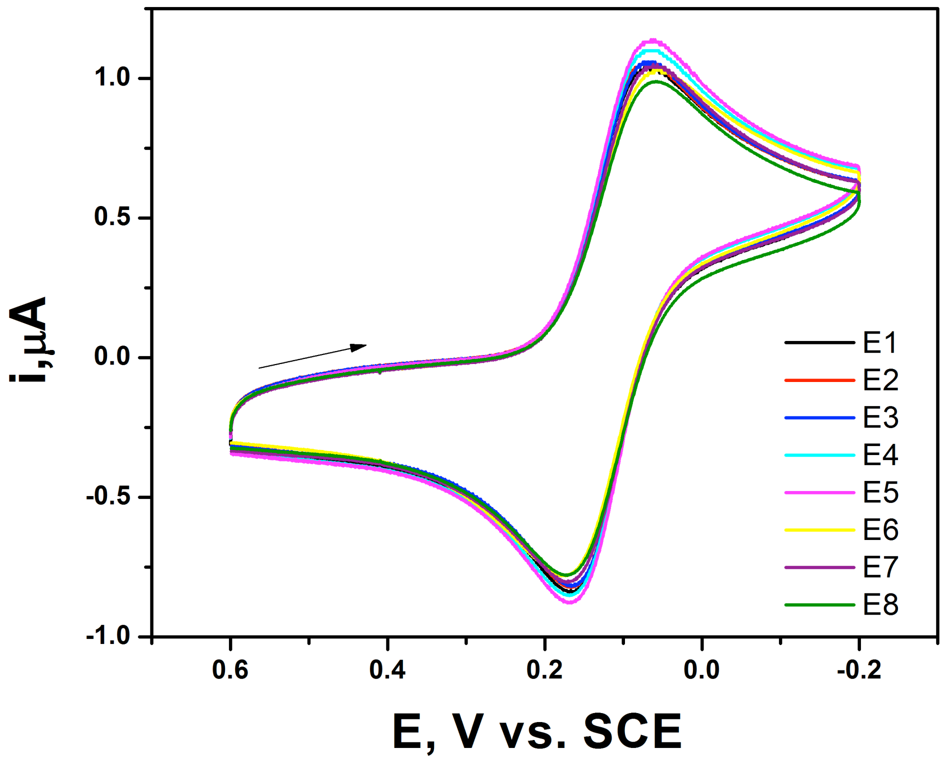

Figure S5: CVs of all eight electrodes simultaneously in an unstirred solution of 1mM potassium ferricyanide solution in 100 mM potassium chloride in the microfluidic device between -0.2 V to 0.6 V at 100 mV s^{-1}.

Prior to each immunoassay, each of the gold arrays were cleaned in 0.18 M sulfuric acid, applying 20 sweep segments between 1.5 V and -0.2 V at 100 mV s^{-1}. Figure S6A shows similar peaks to those found from bulk gold with formation of gold oxide at +1.2 V and reduction back to bulk gold occurring at 0.9 V. [4] From these CVs, the active surface area was calculated following Trasatti *et. al*. [5] to be 0.42 (± 0.02) mm^2 for an individual array with RSD < 5%. This is comparable to the surface area calculated by Jensen *et. al*. of 0.35 ± 0.01 mm^2 in ferricyanide solution using the Randles-Sevcik equation.[1] However, the average surface area calculated over 20 arrays was found to be 0.46 ± 0.1 mm^2, giving an RSD of 22%. Therefore, each amperometric response was surface area corrected due to higher RSD in array-to-array reproducibility. Each elecrodes amperometric response was divided by the surface area calculated from the CVs obtained in 0.18M H_2SO_4 following Trasatti *et. al.* [5] To further assess the reproducibility of the array, amperometric signals were measured on all electrodes in the microfluidic device after injection of 100 mM $Fe(CN)_6{}^{3-/4-}$/ 0.1M KCl. All eight electrodes of the array gave similar peak currents (Figure 6SB), with an average of 2.3 (± 0.13) x 10^{-6} A cm^{-2} (RSD < 6%) demonstrating the minimum cross-talk between neighboring electrodes of the array.

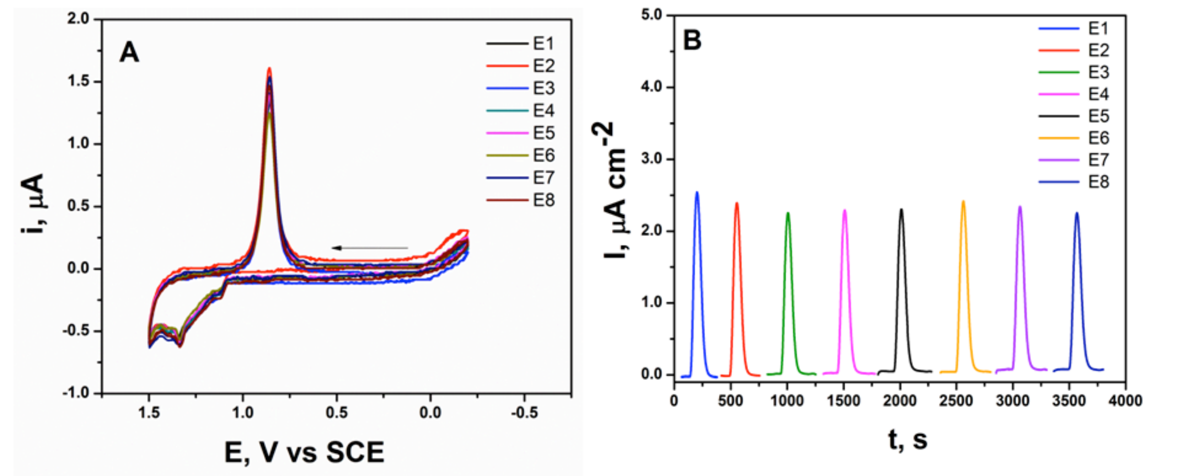

Figure S6: Array reproducibility: (A) CVs at 100 mV s^{-1} on 8-electrodes of the printed gold arrays in 0.18 M H_2S0_4. (B) Amperometric response at 0.1 V vs Ag/AgCl in the microfluidic device after injection of 100 mL of 100 mM $Fe(CN)_6{}^{3-/4-}$ in 100 mM potassium chloride at 100 mL min^{-1}.

Characterization of Array Surfaces after Modification by AFM. Tapping mode AFM images of the working electrode of the Au-arrays after cleaning in 0.18 M *sulfuric acid* revealed a mean surface roughness of 26 ± 2 nm (Figure S7A) which is comparable to that previously reported by Jensen *et. al.* [2] The morphology of the array after the immobilization of Ab_1 had a surface roughness of 13 ± 1 nm (Figure S7B). The globular features demonstrate the immobilization of Ab_1 on the surface with nearly full coverage across the electrode surface. After full immunoassay was performed with the introduction of BSA as well as the bioconjugate (Ab_2-MB-HRP), which resulted in filling the space between the primary antibodies, the surface roughness increased to 32 ± 2 nm with much larger globular features (Figure S7C). The much larger globular features further demonstrate the immobilization of the full immunoassay on the surface.

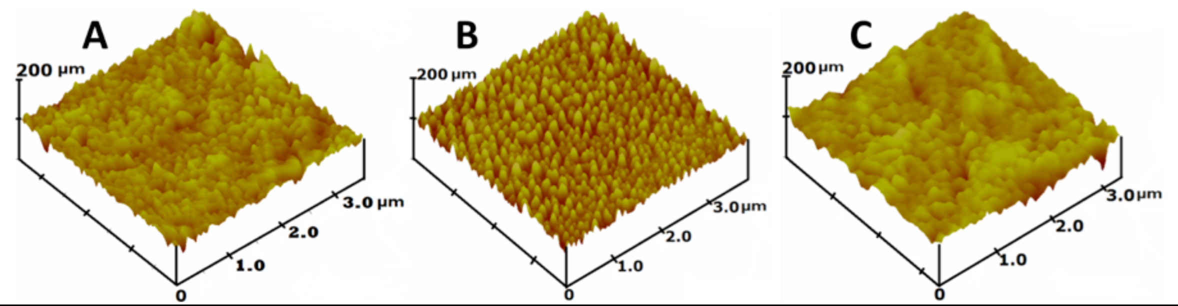

Figure S7: High resolution tapping mode AFM images of one of the eight sensor electrodes of (A) a bare AuNP array after cleaning in 0.18M H_2SO_4 , (B) AuNP array with Ab_1, and (C) AuNP array + Ab_1+ bioconjugate (Ab_2-MB-HRP).

Preparation of Magnetic Ab_2-tosyl or Streptavidin Magnetic Beads (MB)-HRP Conjugates. Ab_2-MB-HRP bioconjugates were prepared as previously described using either *tosylactivated* or *streptavidin*-coated magnetic beads.[6] As previously noted, an Invitrogen Dynal magnet was used to help complete all washing and separation steps required for conjugation of biomolecules to the magnetic beads. Briefly, either 2 mg of *tosyl*-MBs or 0.2 mg of *streptavidin*-MBs were combined with 3 mg HRP and 0.8 mg Ab_2

or 0.2 mg biotin-HRP and 0.8 mg biotin-Ab_2 in a microcentrifuge tube. The total volumes of the conjugation mixtures were ~625 μL for the tosyl-MBs (the bulk of which consisted of a mixture of 3 M ammonium phosphate buffer and 0.1 M sodium borate buffer) and ~200 μL for the *streptavidin*-MBs (beads were suspended in 0.1% BSA in phosphate buffer). The resulting mixtures were incubated at 37°C in an Invitrogen Dynabeads MX mixer for either 24 h (*tosyl*-MBs) or 25 mins (*streptavidin*-MBs). This incubation period facilitated conjugation of HRP and Ab_2 molecules to the bead surfaces. After conjugation, beads were washed with either PBS-T20 (*tosyl*-MBs) or 0.1% BSA in phosphate buffer (*streptavidin*-MBs) to remove excess, unbound HRP and Ab_2. Following washing, *tosyl*-MBs were suspended in 625 μL 0.5% BSA in PBS and incubated for an additional 18 h to remove any remaining unreacted *tosyl* moieties. After sufficient washing in 0.1% BSA in phosphate buffer, Ab_2-MB_{tosyl}-HRP and Ab_2-$MB_{streptavidin}$-HRP were separately suspended in 0.1% BSA in phosphate buffer at 3.2 mg MBs mL^{-1} and 1 mg MBs mL^{-1}, respectively, and stored at 4°C until further use. Both MB conjugates were stable for ~14 days.

Off-line Protein Capture with Tosyl or Streptavidin Magnetic Beads. Previously reported procedures were followed to capture the antigen of interest (IL-6 or IL-8) from standard solutions that were prepared using undiluted calf serum.[1,6] Capture of antigen was performed using 60 μL of MBs for Ab_2-MB_{tosyl}-HRP or Ab_2-$MB_{streptavidin}$-HRP conjugates, respectively, in a microcentrifuge tube. Ab_2-MB-HRP conjugates were mixed with the antigen sample – 5 μL of sample that is diluted with calf serum for either capture using Ab_2-MB_{tosyl}-HRP or Ab_2-$MB_{streptavidin}$-HRP. PBS containing 0.1% BSA was added to bring the total volume of the mixture to ~400 μL. The resulting mixture was then vortexed, placed in a mixer, and incubated at 37°C for 25 mins for capture of the protein analyte using Ab_2-MB_{tosyl}-HRP or Ab_2-$MB_{streptavidin}$-HRP for shorter assay times this step was reduced down to 3 mins. After this time, Ab_2-MB-HRP conjugates with captured antigen were washed twice with 0.1% BSA in PBS to remove any excess unbound antigen. Ab_2-MB_{tosyl}-HRP or Ab_2-$MB_{streptavidin}$-HRP conjugates with captured antigen were then suspended in 160 μL of 0.1% BSA in PBS, and used immediately in immunoassays.

Characterization of Magnetic Bead Conjugates. Using the *bicinchoninic acid* (BCA) protein assay kit (Thermo Scientific, IL, USA) we were able to estimate the number of Ab_2 molecules per MB_{tosyl}, from the amount left in solution after MB_{tosyl}-Ab_2 conjugation and comparing to a standard curve of Ab_2, to be ~110,000 (± 20,000).[1,7][8] For $MB_{streptavidin}$ conjugates the total amount of Ab_2 was estimated by difference using the BCA protein assay kit and found to be ~ 38,000 (± 7,000) Ab_2 molecules per $MB_{streptavidin.}$

The number of horseradish peroxidase labels per magnetic beads for either streptavidin or tosyl was calculated by measuring enzyme activity using *2,2'-Azino-bis(3-Ethylbenzthiazoline-6 Sulfonic Acid)* (ABTS) as reactant. The number of HRPs was measured to be 130,000 (± 35,000) for MB_{tosyl} and 320,000 (± 23,000) for $MB_{streptavidin}$ [1,9]

Table S1: Number of Ab_2 molecules per MB using the *bicinchoninic acid* assay, and number of HRP labels per MB from *2,2'-Azino-bis(3-Ethylbenzthiazoline-6 Sulfonic Acid)* (ABTS) assay.

MP conjugate	Ab_2	HRP
Tosyl-MP	$11\ (\pm 2)\ x10^4$	$13\ (\pm 3.5)\ x\ 10^4$
Streptavidin	$3.8\ (\pm 0.7)\ x10^4$	$32\ (\pm 2.3)\ x\ 10^4$

Fabrication of the Immunosensor. Prior to forming the self-assembled monolayers (SAM) on the surface of the gold arrays with *3-mercaptopropionic acid* (MPA), each of the gold arrays was cleaned in 0.18 M sulfuric acid (S.I. Figure S6A). Once the gold arrays were cleaned, removing any excess oxide species, the arrays were rinsed with water, then ethanol, and finally submerged in 4 mM (MPA) in ethanol for 24 hours. Arrays were then washed with ethanol followed by water and dried with nitrogen gas.

The end carboxylic acids groups from SAM on the array surface were then activated by placing 1 μL of 400 mM EDC and 100 mM NHSS on each electrode (8 electrodes) of the array (Scheme1B). The array was then rinsed with water after 10 mins and primary antibody (Ab_1) was attached by amidization between the activated carboxylic groups of the SAM and the Ab_1 (stock solution of 100 μg mL^{-1}) and left overnight in the fridge at 20^oC. The following morning, arrays were washed with PBS T-20 followed by PBS. This was then preceded by addition of 2% BSA to block nonspecific binding (NSB) on the surface of each array for 1 hour. Once the blocking step was complete, the arrays were again washed with PBS T-20 followed by PBS. Arrays were then ready to be placed in the microfluidic device (Scheme1). The Ab_2-MB-HRP conjugate with off-line captured IL-6 or IL-8 were injected into the microfluidic channel at a flow rate of 100 μL min^{-1}. When the bioconjugate arrived in the channel, as evident by the red-brown MB color, flow was stopped and incubated for 15 mins on the sensor surface (for shorter assay times, this step was reduced to 3 mins). The last step was washing with 0.05%Tween-20 in PBS. Then detection was completed using 1 mM *hydroquinone* (HQ) that was passed through the microfluidic channel at a flow rate of 100 μL min^{-1}. The amperometric signal was generated at -0.2 V vs Ag/AgCl by injecting the mixture of 100 mM H_20_2 and 1 mM HQ in PBS.

Reducing Total Assay Time. In our assay procedures, total assay time was obtained by adding all times after antigen (IL-6 or IL-8) was added. Again, this included the first incubation, in the off-line capture format that was reduced from 25 mins to 10 mins, and finally down to 3 mins, the second incubation, where the conjugate was incubated on the surface of the array, reduced from 15 to 5 and finally to 3 min, along with wash steps and detection that was reduced and took less than 2 mins to perform. We kept a control, containing 0 pg mL^{-1} of IL-6, or IL-8 in serum, and sample, containing 20 pg mL^{-1} (IL-6) or 10 pg mL^{-1} (IL-8) for each of the selected assay times shown in Figure S8A (IL-6), and Figure S8B (IL-8). Therefore, the overall assay time from incubation of sample with Ab_2-MB-HRP to measurement including wash steps was 8 mins.

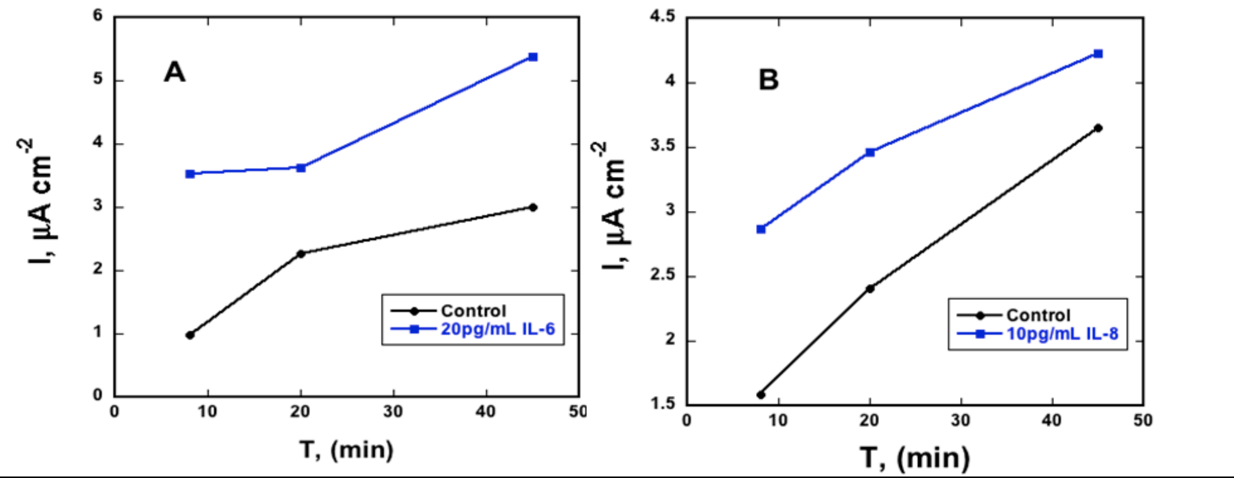

Figure S8: Optimization of assay time for Interleukin-6 reducing the time down to 45, 20, and 8 mins for (A) IL-6 and (B) IL-8.

Assay validation with conditioned cancer cell media. Immunoarray results compared with the ELISA using conditioned media cells (HaCaT, HN12, HN13, and Cal 27) for both IL-6 (figure S8A), and IL-8 (figure S8B). Immunosensor results showed a very good correlation with ELISA for all conditioned media cells with slopes ~1.0 and intercepts ~0, within experimental error.

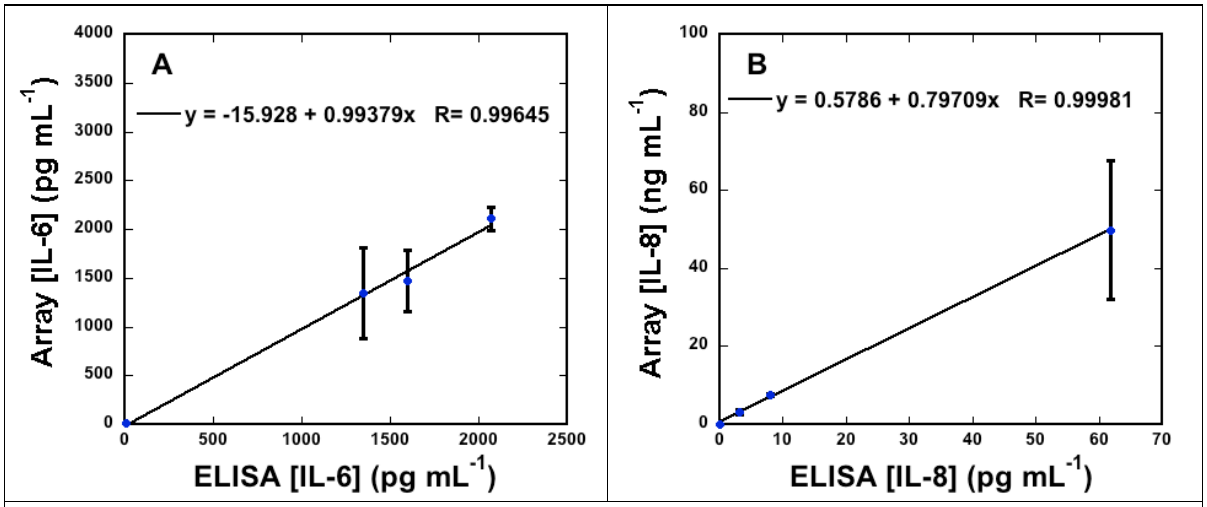

Figure S9: Correlation plots of immunoarray results with standard ELISA for conditioned media for cells (HaCaT, HN12, HN13, and Cal 27) for both IL-6 (A), and IL-8 (B).

Table S2: Slopes and intercepts of correlation plots of immunoarray results for the conditioned media for cells against results obtained from ELISA.

Biomarker	Slope ± sd	Intercept ± sd
IL-6	0.99 ±0.06	-15.9 ± 87.4
IL-8	0.80 ± 0.01	0.58 ± 0.34

References:

[1] B. V Chikkaveeraiah, V. Mani, V. Patel, J. S. Gutkind, J. F. Rusling, *Biosens. Bioelectron.* **2011**, *26*, 4477–83.

[2] G. C. Jensen, C. E. Krause, G. a Sotzing, J. F. Rusling, *Phys. Chem. Chem. Phys.* **2011**, *13*, 4888–94.

[3] S. Sivaramakrishnan, P.-J. Chia, Y.-C. Yeo, L.-L. Chua, P. K.-H. Ho, *Nat. Mater.* **2007**, *6*, 149–55.

[4] S. H. Cadle, S. Bruckenstein, *Anal. Chem.* **1974**, *46*, 16–20.

[5] S. Trasatti, O. A. Petrii, *Pure and Appl. Chem.* **1991**, *63*, 711–734.

[6] R. Malhotra, V. Patel, B. V Chikkaveeraiah, B. S. Munge, S. C. Cheong, R. B. Zain, M. T. Abraham, D. K. Dey, J. S. Gutkind, J. F. Rusling, *Anal. Chem.* **2012**, *84*, 6249–55.

[7] P. K. Smith, R. I. Krohn, G. T. Hermanson, A. K. Mallia, F. H. Gartner, M. D. Provenzano, E. K. Fujimoto, N. M. Goeke, B. J. Olson, D. C. Klenk, *Anal. Biochem.* **1985**, *150*, 76–85.

[8] K. J. Wiechelman, R. D. Braun, J. D. Fitzpatrick, *Anal. Biochem.* **1988**, *175*, 231–7.

[9] J. Putter, In R. Becker, H.U. Bergmeyer, *Methods of Enzymatic Analysis, Verlug Chemie*; Deerfield Beach, FL **1983**, *3*, 286-93.
